# Identification of Factors Associated With School Effectiveness With Data Mining Techniques: Testing a New Approach

**DOI:** 10.3389/fpsyg.2019.02583

**Published:** 2019-11-15

**Authors:** Fernando Martínez-Abad

**Affiliations:** University Institute of Educational Sciences, University of Salamanca, Salamanca, Spain

**Keywords:** data mining, school effectiveness, academic achievement, large-scale assessment, decision trees

## Abstract

The study of school effectiveness and the identification of factors associated with it are growing fields of research in the education sciences. Moreover, from the perspective of data mining, great progress has been made in the development of algorithms for the modeling and identification of non-trivial information from massive databases. This work, which falls within this context, proposes an innovative approach for the identification and characterization of educational and organizational factors associated with high school effectiveness. Under a perspective of basic research, our aim is to study the suitability of decision trees, techniques inherent to data mining, to establish predictive models for school effectiveness. Based on the available Spanish sample of the PISA 2015 assessment, an indicator of the school effectiveness was obtained from the application of multilevel models with predictor variables of a contextual nature. After selecting high- and low-effectiveness schools in this first phase, the second phase of the study was carried out and consisted of the application of decision trees to identify school, teacher, and student factors associated with high and low effectiveness. The C4.5 algorithm was calculated and, as a result, we obtained 120 different decision trees based on five determining factors (database used; stratification in the initial selection of schools; significance of the predictor variables of the models; use of items and/or scales; and use of the training or validated samples). The results show that the use of this kind of technique could be appropriate if mainly used with correctly pre-processed data that include the combined information available from all educational agents. This study represents a major breakthrough in the study of the factors associated with school effectiveness from a quantitative approach, since it proposes and provides a simple and appropriate procedure for modeling and establishing patterns. In doing so, it contributes to the development of knowledge in the field of school effectiveness that can help in educational decision-making.

## Introduction

Identification of educational factors associated with academic performance is a key aspect in educational research into school effectiveness (Rutter and Maughan, 2002; Murillo, 2007; Muijs et al., 2014; Creemers and Kyriakides, 2015). Within this context, we propose an innovative approach to the analysis of good educational practices associated with school effectiveness. This study proposes the application of data mining techniques to identify the main factors that characterize and differentiate high- and low-effectiveness schools.

In contrast to traditionally used techniques (inferential and multivariate correlational statistics), data mining is not based on previous assumptions or theoretical distributions to obtain predictive models. In addition, these techniques are applied with minimal intervention by researchers, which, together with the aforementioned, represent a great advantage for the identification of valuable information in massive databases (Xu, 2005). More specifically, the algorithm proposed in this study is the decision tree (classification algorithm), since it simplifies the analysis and interpretation of the predictor variables and their relationships (Martínez-Abad and Chaparro-Caso-López, 2017).

The main aim of this study, therefore, is the analysis of the fit and predictive power of data mining techniques, specifically decision trees, for the identification of factors associated with school effectiveness in secondary education.

Given this main objective, we can set the following specific objectives:

• Analyze and identify school effectiveness based on cross-sectional data from large-scale assessments.
• Promote methodological alternatives for the study of factors associated with school effectiveness based on mass data.
• Analyze the effectiveness of decision trees (algorithm C4.5) in the study of the process factors associated with school effectiveness.
• Present the possibilities of decision trees for the study of good educational practices in effective schools.

### Conceptual Framework

The publication of the *Equality of Educational Opportunity Study*, better known as the “Coleman Report” (Coleman et al., 1966), had a major impact on the educational research field. The study’s main conclusions were that socio-economic and demographic conditions (contextual factors) provided a decisive explanation of the differences in academic performance between students and schools. It questioned the impact that educational practices carried out in schools could have on student performance. These hard-hitting results fueled an in-depth debate about the contribution of the education system and educational policies to the knowledge and skills acquired by students.

In response to the Coleman Report, the Effective School Movement (ESM) emerged during the 1980s (Lezotte, 1989; Martínez et al., 2009). The ESM began with the aim of identifying and studying the most effective school environments in order to define good educational practices associated with variables over which the education system has control. Since that time, school effectiveness has increased its presence in the educational research field, and today has an important impact on work and scientific dissemination internationally (Rutter and Maughan, 2002; Wrigley, 2013; Muijs et al., 2014; Creemers and Kyriakides, 2015; Chapman et al., 2016; Martínez-Garrido and Murillo, 2016).

In the 1990s, thanks mainly to improvement in the computing capacity of computer systems and to the widespread use of large-scale assessments, research into school effectiveness experienced strong growth and evolution (Chapman et al., 2016). Multivariate statistical analysis based on linear hierarchical models (also called multilevel models) emerged as the fundamental statistical technique for the identification and analysis of school effectiveness (Bryk and Raudenbush, 1992; Creemers and Scheerens, 1994; Goldstein, 1995). These models respect the nested nature of educational data from large-scale assessments and allow the identification of educational groups that show clearly higher or lower performance than expected, taking into account only contextual factors. In this way, based on the study of the residuals of the school in these contextual models, we are able to identify high- and low-effectiveness schools (Gamazo et al., 2018). While some research works propose the carrying out of a qualitative case study of schools identified as high- or low-effectiveness for the analysis of good educational practices (Murillo, 2007; Joaristi et al., 2014), others that are quantitative in nature study these factors by adding to the multilevel models the process variables of interest and analyzing their individual behavior and interaction with other variables (Cordero et al., 2015; Costa and Araújo, 2017; Pitsia et al., 2017; Tan and Hew, 2017). From a methodological point of view, this quantitative perspective faces some problems: the existence of previous assumptions in the analysis that are rarely met or that are not directly taken into account (homocedasticity, normality of distributions, inexistence of non-linear relationships, multicollineality, etc.); difficulties in the estimation of typical errors when dealing with excessively complex models (lack of parsimony) and/or with an excessive number of subjects; and difficulty in studying the multiple interactions between the predictor variables given their high complexity, or the impossibility of doing so when working with fixed-effects models to simplify the computation and interpretation of the data.

That is why this work is interested in proposing a quantitative alternative for the study of process variables associated with school effectiveness that does not have the above-mentioned limitations. Specifically, based on the perspective of educational data mining (EDM), we apply decision trees to establish the predictive models of high- and low-effectiveness schools that have a better fit to the data, and we analyze under which determining factors these techniques achieve better performance.

### Literature Review

The current calculation capacity of computers allows the development and application of appropriate statistical techniques for the analysis of massive data. In this regard, data mining emerges as a set of techniques that add value to large-scale data analysis (Martínez-Abad and Chaparro-Caso-López, 2017). These techniques enable the identification of patterns in the data without proposing previous assumptions or starting models, and with minimal intervention by the researcher (Xu, 2005). Thus, the nature of some of the data mining algorithms, compared to other classic multivariate techniques, can promote significant progress in the identification of factors associated with school effectiveness, guiding decision-making and the operation of the education system at macro, meso, and micro levels (Bronfenbrenner, 1979).

Despite the potential that these statistical techniques may hold, their use in the establishment of performance prediction models in compulsory education is sporadic (Hung et al., 2012; Oskouei and Askari, 2014; S̨ara et al., 2015), and their use for the exploration of large-scale assessments is extremely limited (Liu and Ruiz, 2008; Liu and Whitford, 2011; Kılıç et al., 2017; Asensio et al., 2018). A significant presence of EDM can, however, be observed in the study of performance in university education (Guruler et al., 2010; Kasih et al., 2013; Romero et al., 2013; Kirby and Dempster, 2014; Akçapinar et al., 2015; Tan and Shao, 2015; Asif et al., 2017; Casey and Azcona, 2017; Costa et al., 2017).

Given the characteristics of the statistical techniques of data mining, many of these works focused on non-university levels propose a dichotomous variable as a criterion variable in their models, referring to whether a student reaches a minimum performance (Costa et al., 2017; Kılıç et al., 2017) or if he or she abandons his or her studies (S̨ara et al., 2015). In this regard, although there are data mining techniques that allow the use of ordinal or quantitative criterion variables, and that this dichotomization causes a reduction in the information contained in the original variable (Jacobucci, 2018), the inclusion of dichotomous criterion variables promotes the obtaining of decision trees that have simpler structures and are, therefore, more easily interpretable.

As for the statistical technique applied, although numerous works are carried out with the aim of comparing classification algorithms (Jamain and Hand, 2008; Oskouei and Askari, 2014; Yu et al., 2014; Akçapinar et al., 2015; Asif et al., 2017; Costa et al., 2017; Kılıç et al., 2017), there is no agreement in the scientific community on which ones are more appropriate for the prediction of academic performance. “The literature review suggests that in general there is no single classifier that works best in all contexts to provide good prediction” (Kirby and Dempster, 2014).

In this regard, Jamain and Hand (2008) performed a meta-analysis in which they compared 5807 results of research papers in which classification algorithms were applied. In total, the fit of nine classifiers was compared (linear discriminant analysis, logistic discrimination, kernel methods, naive Bayes, k-nearest-neighbors, CART decision tree, C4.5 decision tree, CN2 rule induction, and multilayer perceptrons). The best fit indices in the models with dichotomous criterion variables were obtained in the decision trees using the C4.5 algorithm.

Oskouei and Askari (2014) established predictive models for the performance of secondary school students based on C4.5 classifiers, Naive Bayes, multilayer perceptrons, RBF neural networks, bagging meta-classifiers, and AdaBoost. Incorporating the educational level of parents contextual variable, the best classifier turned out to be C4.5.

Regarding the application of data mining in the prediction of performance at university levels, in a work by Costa et al. (2017), the C4.5 algorithm achieved the best levels of accuracy in online learning and accuracies similar to the best algorithm (support-vector machine) in classroom learning. In all cases, this algorithm is more accurate than neural networks and naive Bayes. A study by Asif et al. (2017) identified the naive Bayes algorithm as the most accurate in predicting the performance of a university graduate from the initial information provided by the student during enrolment. Other works on university education focus on online learning. A work by Romero et al. (2013) established a predictive model for student performance based on their participation in discussion forums. The naive Bayes classifier and the EM clustering algorithm achieved the best levels of fit, above C4.5. Finally, a study by Akçapinar et al. (2015) analyzed the predictive power of interactions in online environments relating to student performance. Again, the naive Bayes classifier achieved the best levels of fit, slightly higher than those obtained with decision tree C4.5.

In an analysis of large-scale assessments, Kılıç et al. (2017), based on TIMSS 2011 data, established predictive models for mathematics performance based on decision trees (random forest and C4.5), Bayesian networks (naive Bayes), neural networks (multilayer perceptron), and logistic regression. The C4.5 decision tree achieved higher levels of fit than random forest, remaining at levels similar to logistic regression and Bayesian neural networks. For their part, Liu and Whitford (2011) used data from the PISA 2006 assessment to establish predictive models for performance in science. They initially categorized performance in science as a dichotomous variable (satisfactory and unsatisfactory performance), and included predictor variables related to opportunities to learn at home. The levels of accuracy in cross-validation of the models applied were around 70%.

Although the use of data mining has been significantly extended in educational research, no applied works that include a comprehensive study of the stability of the models beyond the report of overfit normally from the cross validation have been found. Stability can be defined as the degree to which an algorithm returns constant results from different samples of the same population (Turney, 1995). Since stability is inversely related to the size of the tree obtained (Jacobucci, 2018), it needs to be studied together with the goodness of fit indices in the analysis of the models.

We should point out that all of the works cited in the state of play use the gross performance of the student as a criterion variable for the predictive models, and only include in a few cases, among their predictor variables, some contextual factors. If we define school effectiveness as “the relation between the observed outcomes and the expected outcomes given the socio-economic context of education systems” (Lenkeit and Caro, 2014, p. 147), we can affirm that these studies do not take into account the fundamentals of the ESM. This argument justifies the proposal that we make in our research. Instead of using gross performance as a study criterion variable, which skews the models in favor of students and schools with higher socio-economic levels (Chapman et al., 2016), we use the identification of the level of effectiveness of the school obtained from the residuals of the schools in the multilevel models applied initially (Gamazo et al., 2018).

## Methodology

Based on an analysis of secondary data from the PISA 2015 assessment (OECD, 2019), this research used a cross-sectional non-experimental research design. To avoid bias in the data related to differences in socio-economic level and the structure of educational systems between countries, we decided to select data from a single country. Spain was the sample selected for several reasons:

• Multilevel models with contextual variables applied to OECD countries based on PISA 2009 data show that Spain is one of the countries with the smallest difference between observed and estimated scores in both reading and mathematics (Lenkeit and Caro, 2014). These results suggest that the Spanish educational system, in relation to other countries assessed in PISA, reaches higher levels of equity. Therefore, data mining models obtained using as criterion variable both gross performance and school-level residuals will be more similar to each other than models obtained in other countries. Given the main objective of this study, it is interesting to be able to compare the fit of the obtained decision trees with other models based on gross performance.
• The size of the Spanish sample in PISA 2015 was much larger than that of most of the sampled countries since each of its 16 autonomous communities is taken as a stratum.

### Participants

Taking into account the aforementioned, our starting point was the population of Spanish students who at the time of the 2015 PISA assessment were 15 years old, their teachers, and the schools in which they studied. In Spain, students who had undergone standardized schooling were at that time in the final year of compulsory secondary school.

From this population, the initial sample obtained was 32,330 students, 4286 teachers, and 976 schools. However, to obtain more stable estimates of the aggregated variables at the school-level (obtained from the calculation of the average score of the first level variables), and to get better estimates of multilevel model parameters, we removed from the sample all schools with less than 20 students (Hox, 2010; McNeish and Stapleton, 2016). After this filtering, the sample on which the multilevel models of the first phase of the research were applied had 871 schools, 31,105 students, and 3682 teachers.

As will be discussed later, the results of the multilevel models enabled the selection of high- and low-effectiveness schools.

The sample weights proposed in the PISA 2015 data served to weigh the data in both phases.

### Variables and Instruments

The instruments included in the 2015 PISA tests, which we used in this study, were obtained from two sources:

• Performance tests in reading, mathematics, and science. The PISA tests used a sampling of items from which the ability of each student in the three areas was estimated using the item response theory (IRT). Thus, PISA assessment includes an estimate of 10 plausible values of the achievement of each student in the three main assessed areas.
• Questionnaires used with management teams (school information), teachers, and students. These questionnaires included abundant information on socio-economic context, educational processes and organizational issues, cognitive and personal aspects of students, etc.

While the reliability and validity of the achievement tests included in PISA are evidenced extensively in the technical reports (OECD, 2017), with there being general agreement in the academic community about their relevance (Hopfenbeck et al., 2018), this same level of agreement does not exist in relation to the context questionnaires obtained at student, teacher, and school level (Rutkowski and Rutkowski, 2010; González et al., 2012; Fernandez-Cano, 2016; González-Such et al., 2016). Although estimation of the dimension scores is obtained from psychometric procedures based on IRT, lower reliability is observed on these estimates, evidenced by the low correlation between the responses of students and families on similar matters (Rutkowski and Rutkowski, 2010). We should not forget that the context questionnaires in PISA include several self-perception scales and self-report measurements. Thus, there are broad criticisms on several fundamental matters:

• Social, cultural, and economic significance of the defined constructs: Cultural differences between countries make it difficult to compare the significance of these constructs and, therefore, to make cross-cultural comparisons (Hopfenbeck et al., 2018).
• Lack of stability in the definition of indicators, items, and constructs: Several items and scales change from one edition to another, others are discarded, and some others are included (Fernandez-Cano, 2016).
• Poor translations of the questionnaires into languages other than English: The versions of these questionnaires (including in this case the achievement measurement tests) in the different languages make their comparability difficult (Huang et al., 2016).
• Missing data: Contrary to what happens with the achievement measurements, which rarely include missing data in the student database, the measurements and constructs of the context questionnaires include missing data on a regular basis (Hopfenbeck et al., 2018).

As a result, although the OECD is making significant efforts in the latest editions of PISA for the improvement of these aspects (Jornet, 2016), we need to be cautious when interpreting the results obtained from these scales in their transfer to educational policies.

Regarding variables, the following were used:

• In the application of the multilevel models, the criterion variables used were gross performance in the three areas assessed at student level (Level 1) and the average performance of the school (Level 2). Unfortunately, although a teacher database is included in PISA 2015, we could not include classroom-level variables in the models since these data do not allow to associate teachers with students in their classroom.
• The predictive variables used, which were exclusively contextual in nature, were the following:
  ∘ Level 1: Gender; birth month; academic year; index of economic, social, and cultural status (ESCS); migratory status; repetition of academic year; number of school changes; mother tongue.
  ∘ Level 2: Size of the school; classroom size; shortage of resources; shortage of teachers; school ownership; student/teacher ratio; average ESCS; repeater rate; immigrant student rate; proportion of girls.
• The decision trees included as a criterion variable the identification of the school as high or low effectiveness (dichotomous variable). The predictor variables included in the decision trees were all non-contextual items and scales included in the PISA 2015 databases, both in schools and in teachers and students. In total, the decision trees used included 232 variables (39 of teachers, 139 of students, and 54 of school).

Selection of the variables included in the multilevel models draws from the focus of this research, which is based on the context-input-process-output (CIPO) model. This model (Creemers and Scheerens, 1994; Creemers and Kyriakides, 2015) raises the need to differentiate between the variables on which schools and their educational communities can exert influence (process variables) and those on which it has no decision-making power (context and input variables). Thus, while context variables refer to the socio-economic and cultural environment that surrounds the school and its members, input variables are related to the personal and economic resources available and to the background of the students. From this categorization of variables, it is possible to speak about two types of school effects (Raudenbush and Willms, 1995): the first, or type-A effects, are defined as the difference between the achievement of a student and what would be obtained if he or she went to a school with certain contextual characteristics; the second, or type-B effects, can be defined as the difference between the achievement of a student in a certain school and what is expected to be obtained if that student attended a school with the same contextual conditions but with different procedural conditions (school organization, teaching methodologies, leadership process, decision-making, etc.). Thus, in the multilevel models applied in the first phase of this study, type-B effects (residuals of Level 2 of the models) are tried to be detected after controlling the type-A effects (by introducing the input and context variables as co-variables in the models).

In particular, selection of the context and input variables used in the multilevel models is based on the literature review carried out both theoretically (Creemers and Scheerens, 1994; Chapman et al., 2016) and from previous studies in similar contexts (Murillo, 2007; Joaristi et al., 2014).

### Procedure and Analysis of the Data

HLM7 software was used to calculate the multilevel models, which were applied taking into account the 10 plausible values provided by PISA 2015 in each of the three areas assessed. HLM 7 computes an independent model for each of the available plausible values and returns the parameters averages. Since HLM7 does not allow the use of sample replicate weights, to minimize bias in error estimation this software employs robust estimators using the Huber-White adjustment. This adjustment compensates for the biases associated with the omission of replicate weights (Lavy, 2015; Lopez-Agudo et al., 2017).

From a significance level of 5%, we included only significant predictor variables in the multilevel models. Since the three models obtained from each achievement measurement were clearly different in terms of the predictor variables included (Gamazo et al., 2018), we consider it more appropriate to calculate three independent models.

Finally, we computed the significant models, with random intercepts and fixed slopes in school level, and calculated the residuals of the school level using empirical Bayes estimation (Raudenbush et al., 2016). In all of the cases, the values we obtained in the intraclass correlation coefficient (ICC) were greater than 10% in the null models (Eq. 1), considered as the minimum acceptable value to consider multilevel methods (Roberts et al., 2011):

(1) $$y_{\mathrm{ij}}=\gamma_{00}+u_{0\,j}+e_{\mathrm{ji}}$$

where *y*_*ij*_ refers to the performance achieved by student *i* of school *j* in the corresponding area. Thus, in the null models, *y*_*ij*_ represents the sum of the overall average performance in the corresponding area (γ_00_), the distance of average school performance *j* from the overall (*u*_0_*_*j*_*), and the distance of student performance *i* with respect to the school *j* (*e*_*ji*_).

The final models obtained in each area are specified in Eq. 2:

(2) $$y_{\mathrm{ij}}=\gamma_{00}+\sum_{s=1}^{S}\left(\gamma_{0\,s}\,W_{\mathrm{sj}}\right)+\sum_{q=1}^{Q}\left(\gamma_{\mathrm{qj}}\,X_{\mathrm{qji}}\right)+\left(u_{0\,j}+e_{\mathrm{ji}}\right)$$

where γ_0_*_*s*_* and γ*_*qj*_*, respectively, represent the main effects on the variables of school and student level, *W*_*sj*_ variables *s* of school, *j*, and *X*_*qij*_ variables *q* of student *i* of school *j*.

After obtaining the residuals of the schools in the three final models, we carried out the selection of high- and low-effectiveness schools. To do so, we carried out a first selection of schools (non-stratified selection), in which the schools that were placed in the first quartile in the three computed models (schools of negative residual, low effectiveness) and the schools that were placed in the last quartile in the three models (positive residual schools, high effectiveness).

Given the extensive educational competence of the autonomous communities in Spain, we made a second selection of schools (stratified selection). In this second case, we used the same criteria indicated above in each of Spain’s 16 autonomous communities, implementing 16 separate selective processes for high- and low-effectiveness schools. The residuals used in this selection were the same residuals used in the original selection. We opted for this procedure because ICC levels were below 10% in the null models of the specific samples in some autonomous communities. The decision to create a dichotomous variable from the residuals obtained in school level addresses two fundamental questions:

• The use of dichotomous criterion variables in obtaining decision trees simplifies the interpretation of the rules obtained in the models.
• The residuals used are indicators with estimation errors associated with them, so the use of their absolute values is not appropriate.

The decision trees were computed using Weka 3.8.1 free software. Given the results shown in the state of play, we decided to use the C4.5 algorithm in the construction of the models. This algorithm is an extension of ID3 (Quinlan, 1986, 1992), and its use is widespread in EDM to model student performance (Martínez-Abad and Chaparro-Caso-López, 2017; Rodrigues et al., 2018). The fit of this algorithm allowed the use of variables of all types (categorical and scale), and uses the information gain ratio for their selection. This facilitates the computation of simple models (Quinlan, 1986). With the intention of obtaining reduced trees and avoiding problems of readability and overfitting, we decided to limit the maximum number of branches to 30 (Kieskamp, 2015) to ensure that the trees obtained were easily interpretable and not overfitted. The overall fit of the decision trees was assessed based on true positive (TP), accuracy (percentage of correctly classified instances), area under the ROC curve, and kappa indicators. According to previous studies (Mitchell, 2009; Martínez-Abad and Chaparro-Caso-López, 2017), 70% of correctly classified instances were established as the cut point to determine an acceptable fit index.

As an additional control measure, we include the study of the stability of the trees obtained (Jacobucci, 2018). Taking into account that the variance of the cross-validation accuracy estimators is higher if the algorithm is unstable (Liu and Motoda, 1998), we will evaluate the internal stability of the decision trees (Aluja-Banet and Nafria, 2003) from the standard deviation and the coefficient of variation of the accuracy levels obtained in cross validations with 100-folds.

To take a known point of reference that allows the fit level of the models obtained to be assessed, logistic regression models are applied. Selection of the predictor variables included in these models is automated through the use of the LogitBoost algorithm (Landwehr et al., 2005).

It was necessary to generate a total of 120 different databases based on different determining factors:

• Informant of predictor variables (*Database*—five categories): Predictor variables from the student database; predictor variables from the teacher database; predictor variables from the school database; student and school variables with student scores aggregated at school level (aggregate data); student and school variables with the school scores included in the student level (non-aggregate data).
• Type of predictor variables included (*Items-scales*—three categories): Only items, not including scales; only scales; both items and scales.
• Stratum by Spanish region (*Stratum*—two categories): Identification and selection of high- and low-effectiveness schools in one step from the complete sample of schools in Spain; identification and selection of high- and low-effectiveness schools independently in each of the 16 regions, taking into account stratification by region.
• Significance level of the predictor variables (*Significance*—two categories): All predictor variables, both significant and non-significant, were included; only significant predictor variables were included.
• Type of sample to obtain the model (*Validation*—two categories): The models were computed from the training sample; the models were computed from a cross-validation with 10 sub-samples.

This made it possible to compute a total of 120 different decision trees based on these five determining factors (for example, one of the 120 trees calculated included as predictor variables the significance scales of the student database and as a criterion variable the identification of high- and low-effectiveness schools taking into account stratification by region, estimating the fit indices from the training sample).

After this process, we were able to compare the fit of the trees obtained based on these five determining factors. To do so, we calculated the average scores and typical overall deviations and by interest groups and used the appropriate hypothesis contrasts to compare the groups. This procedure made it possible to identify the categories or groups with best and worst fit in the predictive models.

## Results

### Multilevel Models

The initial ICC in the three models applied was acceptable (science = 12.41%; reading = 12.04%; mathematics = 12.26%). The ICC of the final models achieved acceptable levels (science = 5.60%; reading = 5.07%; mathematics = 4.55%), since in the three models the variance explained at school level accounted for more than 50% of the total variance. The most explanatory model was the competence in mathematics predictor, in which the predictor variables accounted for 62.99% of the total variance of the second level.

The breakdown of selected schools, based on the two procedures described in the methodology, can be seen in Table 1. The non-stratified selection method returned a larger and more balanced sample of high- and low-effectiveness schools, while, with the stratified method, more low-effectiveness schools were selected. In no case did the selection of a school by one of the methods produce an inverse result to the other method.

**TABLE 1:** Breakdown of high- and low-effectiveness schools according to the selection procedure.

	**Not stratified**			
	**High**	**Not selected**	**Low**	**Total**
**Stratified**				
High	75	34	0	109
Not selected	55	518	59	632
Low	0	62	68	130
Total	130	614	127	871

Table 2 shows the breakdown of the student and teacher sample according to school selection based on stratification or without taking it into account. In this case, the use of the two methodologies returned a sample with a similar breakdown in terms of size in high- and low-effectiveness schools.

**TABLE 2:** Breakdown of students and teachers according to selection procedure.

		**Not stratified**			
		**High**	**Not selected**	**Low**	**Total**
**Stratified**					
High	Students	2569	1211	0	3780
	Teachers	294	58	0	352
Not selected	Students	2016	18,553	2230	22,799
	Teachers	241	2206	340	2787
Low	Students	0	2198	2328	4526
	Teachers	0	141	402	543
Total	Students	4585	21,962	5448	31,105
	Teachers	535	2405	742	3682

### Decision Trees: Stability and Fit

The average accuracy levels obtained according to each of the determining factors are shown in Table 3. Under parentheses are presented the accuracy levels obtained in the logistic regression models. Regarding *Database*, it can be observed that the highest levels of accuracy, both in the training and validated samples, are found in the samples that include school and student data, with them being higher in the aggregate data of the training sample and non-aggregate data of the validated sample. Except in the aggregate data, the models predict low effectiveness better. No significant differences were obtained regarding the use of *items and scales*. While the validated sample was more accurate if data with *significant variables* were used, in the training sample, trees in which significant and non-significant variables were used were slightly more accurate. Finally, slight differences were obtained in the training sample in terms of the use of *stratified* or *non-stratified* selection, which increased in the validated sample in favor of the stratified sample. In general, better predictive levels were maintained for low-effectiveness schools in both the training and validated samples.

**TABLE 3:** Average accuracy of the decision trees according to determining factors (under parentheses accuracy of logistic regression models).

		**Training**			**Validated**		
		**Total**	**High**	**Low**	**Total**	**High**	**Low**
DDBB	Schools	0.803 (0.767)	0.801 (0.767)	0.812 (0.768)	0.502 (0.526)	0.483 (0.506)	0.518 (0.539)
	Teachers	0.665 (0.665)	0.647 (0.612)	0.676 (0.677)	0.581 (0.630)	0.453 (0.523)	0.610 (0.652)
	Students	0.626 (0.631)	0.617 (0.617)	0.633 (0.641)	0.591 (0.604)	0.578 (0.587)	0.603 (0.618)
	Aggr. school + student	0.934 (0.950)	0.943 (0.951)	0.927 (0.950)	0.717 (821)	0.718 (0.809)	0.717 (0.833)
	Not Aggr. school + student	0.807 (0.834)	0.786 (0.829)	0.846 (0.838)	0.786 (0.823)	0.781 (0.819)	0.797 (0.828)
Items-scales	Items + scales	0.773 (0.811)	0.759 (0.800)	0.788 (0.817)	0.638 (0.704)	0.609 (0.680)	0.650 (0.717)
	Items	0.769 (0.802)	0.758 (0.792)	0.777 (0.808)	0.632 (0.706)	0.604 (0.682)	0.642 (0.720)
	Scales	0.760 (0.695)	0.759 (0.675)	0.772 (0.700)	0.636 (0.633)	0.594 (0.585)	0.654 (0.645)
Significance	All	0.772 (0.808)	0.763 (0.795)	0.782 (0.814)	0.633 (0.704)	0.597 (0.677)	0.649 (0.716)
	Significant	0.762 (0.731)	0.754 (0.715)	0.775 (0.736)	0.637 (0.658)	0.608 (0.621)	0.648 (0.672)
Stratum	Stratified	0.776 (0.777)	0.768 (0.757)	0.784 (0.783)	0.649 (0.687)	0.597 (0.637)	0.673 (0.706)
	Not stratified	0.758 (0.762)	0.749 (0.754)	0.773 (0.767)	0.621 (0.675)	0.608 (0.661)	0.625 (0.682)

Although the level of general accuracy of the logistic regression models is slightly higher, mainly in the validated data, we should bear in mind that a maximum number of predictor variables is not set in these models. It should be remembered that a maximum size of 30 branches is set in the decision trees. Thus, while the logistic regression models reach an average number of 47.12 predictor variables included (reaching a maximum of 174 variables in one of the models), the decision trees feature, on average, 15.10 rules (with a maximum of 30 rules). Therefore, this fact significantly affects the fit levels of the models.

Table 4 shows an analysis of the stability of the decision trees obtained. In general, average instabilities slightly below 40% are obtained, meaning that the level of accuracy of each repetition of the cross validation of a tree, on average, is 40% away from average accuracy. The only factor in which significant differences are found is in the databases, where it is observed that non-aggregate student and student + school data are noticeably more stable, while, in school data, high levels of instability are reached.

**TABLE 4:** Stability of obtained decision trees (100-folds cross-validation) according to determining factors.

		**Accuracy**	**SD**	**CV**
DDBB	Schools	0.502	0.363	69.76%
	Teachers	0.581	0.156	26.61%
	Students	0.591	0.077	12.83%
	Aggr. school + student	0.717	0.315	44.18%
	Not Aggr. school + student	0.786	0.058	7.30%
Items-scales	Items + scales	0.638	0.257	39.99%
	Items	0.632	0.249	38.48%
	Scales	0.636	0.247	38.57%
Significance	All	0.633	0.251	38.63%
	Significant	0.637	0.250	39.38%
Stratum	Stratified	0.649	0.251	37.90%
	Not stratified	0.621	0.250	39.99%

In general, decision trees were obtained with highly variable levels of fit depending on the determining factors proposed (Table 5). Certain acceptable average accuracy and TP indices can be observed in the training sample both in total prediction and the prediction of high- and low-effectiveness schools. The models were, however, significantly more accurate for low-effectiveness schools. The average fit indices in the validated data were insufficient, although, in the maximum values, acceptable scores were achieved.

**TABLE 5:** Overall fit of the decision trees proposed.

		**High effectiveness**		**Low effectiveness**		**Global**	
		**(Min, Max)**	**Mean (*SD*)**	**(Min, Max)**	**Mean (*SD*)**	**(Min, Max)**	**Mean (*SD*)**
TP	Training	(0.18,0.95)	0.697 (0.208)	(0.59,0.97)	0.815 (0.103)	(0.59,0.95)	0.767 (0.114)
	Validated	(0.14,0.88)	0.552 (0.187)	(0.40,0.91)	0.698 (0.124)	(0.40,0.87)	0.635 (0.116)
Accu.	Training	(0.56,0.97)	0.756 (0.125)	(0.61,0.95)	0.779 (0.113)	(0.59,0.95)	0.767 (0.114)
	Validated	(0.32,0.88)	0.602 (0.146)	(0.40,0.88)	0.649 (0.116)	(0.40,0.87)	0.635 (0.116)
Kappa	Training	–	–	–	–	(0.18,0.90)	0.515 (0.246)
	Validated	–	–	–	–	(-0.20,0.76)	0.248 (0.247)
ROC	Training	–	–	–	–	(0.62,0.98)	0.802 (0.122)
	Validated	–	–	–	–	(0.38,0.92)	0.652 (0.135)

**FIGURE 1:**
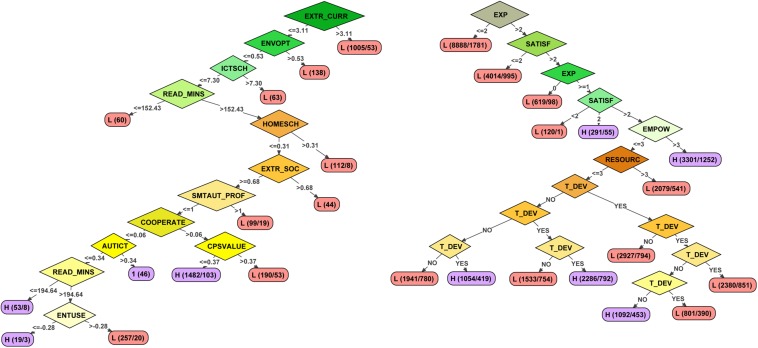
Example of two trees obtained in the study.

An example of two of the very different decision trees obtained in this study is presented in Figure 1. It can be observed that the models are simple and intuitive. The tree on the right was the one obtained from the following determining factors: Aggr. School + Stud, Scales, All, Stratified. The accuracy obtained was 0.93 in the training sample and 0.87 in the validated sample, with very similar accuracy in the prediction of high- and low-effectiveness schools. The second tree was obtained from the following factors: Teachers, Items, Significant, Stratified. It obtained an accuracy of 0.71 in the training sample (0.63 in the validated sample). In this case, accuracy in predicting low-effectiveness schools is around 10 points higher than prediction of high effectiveness.

The rhombuses show the predictor variables included in the models and the ellipses the accuracy of each rule established by the tree. While the arrows provide information on the range of scores of the previous variable included in the rule, each ellipsis provides information on the level of accuracy of the rule (the first letter represents the prediction of schools that meet that rule as high or low effectiveness, the first number indicates the number of elements included in the rule, and the second the elements whose level of effectiveness does not match the prediction). Thus, in the right-hand tree, it can be observed that very low levels of teacher experience and job satisfaction are the fundamental variables that predict low effectiveness. Meanwhile, in schools that have teachers with higher levels of experience and job satisfaction, it is also necessary to have a staff committed to decision-making in the school and to their own teaching development.

### Decision Trees: Accuracy Comparisons

Comparison of the fit of the models according to the determining factors was carried out in the validated samples through the ANOVA test, using accuracy as a dependent variable. Therefore, the determining factors *Database*, *Items-scales*, *Stratum*, and *Significance* were compared, including all of these variables as fixed factors and only the significant interactions. In Table 6, we can see the result obtained for the overall accuracy of the decision trees. The model obtained achieved a very high general fit (adjusted *R*^2^ = 89.4%). Significant determining factors were observed in *Database* and *Stratum*, with a very high effect size in the first. Significant interactions resulted between *Database* and *Significance*, *Database* and *Stratum*, and *Significance* and *Stratum*. The first two interactions achieved average effect sizes.

**TABLE 6:** Decision tree fit comparison ANOVA table—overall accuracy.

	***F***	***p***	**η^2^**
Intercept	16, 860.041	<0.001	0.998
DDBB	108.829	<0.001	0.912
Items-scales	0.154	0.857	0.007
Significance	0.140	0.710	0.003
Stratum	7.997	0.007	0.160
DDBB^∗^significance	8.902	<0.001	0.459
DDBB^∗^stratum	6.979	<0.001	0.399
Significance^∗^stratum	6.186	0.017	0.128

**FIGURE 2:**
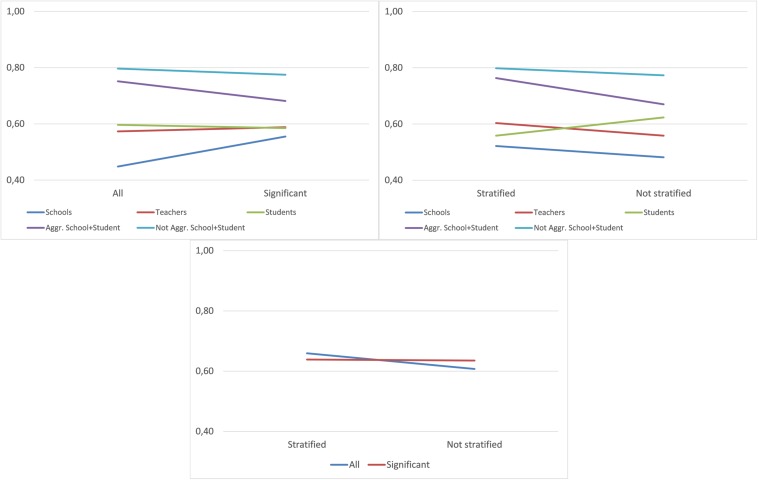
Interaction between determining factors—overall accuracy.

Figure 2 shows the average accuracy in data divided by the interaction variables. Taking the school and teacher data, we can observe that the trend is different to the rest of the data, which includes student information. When student information is included, it seems preferable to use the data with all of the variables, while with data from teachers and, mainly from schools, the use of significant variables improves the accuracy of the models. If we compare *Database* with *Stratification*, we observe that it is preferable to use stratified data, unless we have data exclusively from students, in which case the improvement is significant in the non-stratified sample. Finally, a slight interaction between *Significance* and Stratum can be observed: With stratified data, the incorporation of all variables is preferable.

Table 7 shows the results obtained in the models for accuracy in high- and low-effectiveness schools, respectively. Significant determining factors varied in both models. While *Database* was significant in both cases with large effect sizes, *Stratum* was only significant in the case of low effectiveness, with a medium–low effect size. Significant interactions resulted between *Database*–*Significance* and *Database*–*Stratum*. The models achieved a high overall fit both in the high-effectiveness model (adjusted *R*^2^ = 87.8%) and in the low-effectiveness model (adjusted *R*^2^ = 83.6%).

**TABLE 7:** Decision tree fit comparison ANOVA table—high and low effectiveness.

	**High effectiveness**			**Low effectiveness**		
	***F***	***p*.**	**η^2^**	***F***	***p*.**	**η^2^**
Intercept	8372.433	<0.001	0.995	11, 456.530	<0.001	0.996
DDBB	95.475	<0.001	0.899	64.474	<0.001	0.857
Items-scales	0.449	0.641	0.020	0.339	0.714	0.016
Significance	0.656	0.422	0.015	0.012	0.913	<0.001
Stratum	0.741	0.394	0.017	15.683	<0.001	0.267
DDBB^∗^ significance	6.573	<0.001	0.379	5.388	0.001	0.334
DDBB^∗^ stratum	7.347	<0.001	0.406	5.021	0.002	0.318

**FIGURE 3:**
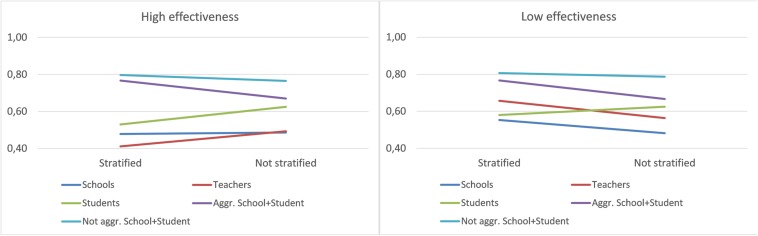
Interaction between Database and Significance—high and low effectiveness.

The significant interaction between Database and Significance is further analyzed in Figure 3. We observe an interaction with a trend similar to that obtained in the overall data. However, in the low-effectiveness data, teachers achieved a better fit, with a similar fit in the data with significant variables and all variables.

**FIGURE 4:**
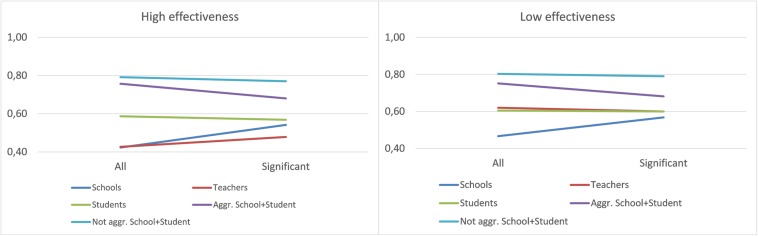
Interaction between Database and Stratum—high and low effectiveness.

Finally, Figure 4 presents the graphs of interaction between *Database* and *Stratum*. While the trend in low effectiveness was similar to the overall one, in high effectiveness, differences were observed: In the teacher data, the use of stratified data improved the accuracy, and in the school data, no great differences were observed between the two *Stratum* cases.

## Discussion and Conclusion

The main aim of this work was to study the relevance of the use of decision trees for the study of educational factors associated with school effectiveness using data from large-scale assessments. We decided to use the C4.5 algorithm since it allows the use of variables of all kinds (Quinlan, 1986) and facilitates the obtaining of simple and easily interpretable models (Martínez-Abad and Chaparro-Caso-López, 2017). In addition, evidence from previous studies shows that it is an algorithm with highly satisfactory levels of fit both in the study of secondary school performance (Oskouei and Askari, 2014) and in the prediction of performance in large-scale assessments (Kılıç et al., 2017), higher than other classification algorithms when the criterion variable is dichotomous (Jamain and Hand, 2008). The results obtained allowed us to affirm that the fundamental aim proposed in the work was satisfactorily fulfilled thanks to the analysis performed.

On the one hand, a descriptive study of the level of fit of the decision trees was carried out based on several important determining factors. The results seem to indicate that the factor that creates the most differences in the accuracy achieved and in the stability is the *Database* used. As expected by the increase in the number of available variables, it seems that the use of school and student data combined produces better levels of fit. Observing the validated models, it seems that the use of non-aggregate data is preferable since it returns results with no overfitting in the training sample. For its part, as the previous evidence pointed out (Martínez-Abad and Chaparro-Caso-López, 2017), school data show high overfitting in the training sample. According to this evidences, stability levels were higher when students were established as the unit of analysis. Both the inclusion of significant variables and the selection of high- and low-effectiveness schools based on the stratification of the sample also result in less overfitted data, with better fit indices in the validated sample. These results coincide with the state of play, which shows significant improvements in the fit of the models when adequate pre-processing of the data is performed (Romero et al., 2013; Costa et al., 2017).

Taking into account that in this study decision trees with a very small size (maximum 30 branches) were selected, the levels of average accuracy achieved, which were above 0.76 in the training sample, were satisfactory. Other previous works that did not limit the size of the decision trees achieved overall accuracy levels of between 0.7 and 0.8 (Oskouei and Askari, 2014; Akçapinar et al., 2015; Costa et al., 2017; Kılıç et al., 2017). The study by Liu and Whitford (2011) is particularly interesting since they developed a predictive model of performance from the data of students in PISA 2006 using the C4.5 algorithm. They obtained a tree with more than 100 branches, achieving an accuracy in the validated sample (10-fold cross-validation) of 0.70. Our results achieved similar levels of accuracy using trees of a significantly smaller size and from a criterion variable to which the variability of contextual factors was previously eliminated.

Also notable in the results obtained was the superior fit of the models for the prediction of low-effectiveness schools. The calculated models achieved a TP rate almost 15% higher in these schools than in those of high effectiveness in the validated results (and 5% higher accuracy). Since other works seem to point to this trend based on more applied analysis (Martínez-Abad et al., 2018), it is worth delving deepen into its explanation and practical implications.

In addition, an inferential study was carried out both on the significance of the main effects of the determining factors analyzed and on the interactions between these factors. The results reflected those indicated above, highlighting that the strongest determining factor in the accuracy of the models was *Database*. *Stratum*, the other significant factor identified, reflected the importance of performing good pre-processing of the data. In contrast to the works of Romero et al. (2013) and Costa et al. (2017), in which a prior reduction in the number of variables produced an improvement in the fit of the predictive models, in our study, this factor did not achieve significant main effects. This difference may have to do with the criterion variable used in our study, which was qualitatively different to gross performance.

The interactions studied show interesting trends not assessed in previous works: on the one hand, *Databases* with better fits work worse when only significant variables are used as predictors. These effects are maintained in the independent prediction of high- and low-effectiveness schools. Regarding the interaction with *Stratum*, the student database is the only one with better levels of fit with non-stratified data. In high-effectiveness schools, however, teachers show this same trend. There is no clear pattern in these last results, and a more detailed study of them is necessary.

Several consistent strengths and contributions in our study are, therefore, confirmed, mainly in relation to the good fit shown by a good number of the models applied in general, and especially by one of them in particular. It seems that the use of decision trees from correctly pre-processed data, which includes abundant and combined school, teacher, and student information, returns predictive models of school effectiveness with good fits both in the training and validated samples. Some weaknesses inherent to this work should, however, also be highlighted. On the one hand, we find the restrictive nature of the categorization performed in the criterion variable to obtain a dichotomous variable. This decision eliminates much of the variability of this variable, limiting the possibilities of pattern identification in the data. In this regard, we decided to prioritize the obtaining of easily interpretable decision trees over trees that are very tight fitting but difficult to apply to educational reality and decision-making. We believe that this is the most appropriate procedure to facilitate the transfer of the results obtained given the level of development and current possibilities of the techniques used. In this sense, we must also point out that the computation of small trees, easily interpretable, could make it difficult to obtain trusted trees (Jacobucci, 2018). On the other hand, a more in-depth study of the implications of using aggregate data from the averages for the use of classification algorithms, or incorporating data from higher levels (school) to databases of lower levels (students) should be carried out. Comparison of the fit obtained in the database achieved by each of these two procedures shows that both methodologies have their advantages and disadvantages, with it being necessary to clear up any uncertainties about the biases that go with each one of them. Finally, it should be noted that in the first phase of the study, three independent multilevel models were calculated, one for each subject assessed by PISA, so the calculations do not take into account the covariance between the three achievement variables. Although this decision is justified by the clear differences between the variables included in the three final models, future studies could focus on the obtaining of models that integrate all of the information from the three dependent variables (e.g., from MIMIC models).

Many future research lines of great interest for the educational scientific community, mainly in two areas, are therefore opened up for the near future. Regarding the carrying out of more basic studies similar to this one, we need to increase the volume of evidence and contributions, since there are no similar studies that use school effectiveness as a criterion variable: Works that compare the operation of various classification algorithms; systematic analysis of the implications of using combined aggregate and non-aggregate data; and studies similar to this one in which the gross residual of school effectiveness (scale variable) or politomic categorization is used. With respect to the use of studies closer to that used, there is an undeniable potential regarding the use and interpretation of specific decision trees in various databases to try to identify factors associated with effectiveness, thereby contributing to the educational characterization of high- and low-effectiveness schools.

## Data Availability Statement

The datasets for this study can be found in the OECD webpage: https://www.oecd.org/pisa/data/2015database/.

## Ethics Statement

Ethical review and approval was not required for the study on human participants in accordance with the local legislation and institutional requirements. Written informed consent to participate in this study was provided by the participants’ legal guardian/next of kin. This study is based on the public databases of the PISA 2015 assessment (OECD). Data collection for OECD-PISA studies is under the responsibility of the governments from the participating countries.

## Author Contributions

This work was completely conducted and developed only by FM-A.

## Conflict of Interest

The author declares that the research was conducted in the absence of any commercial or financial relationships that could be construed as a potential conflict of interest.

## References

[B1] Akçapinar G. Altun A. As̨kar P. (2015). Modeling students’ academic performance based on their interactions in an online learning environment. *Elem. Educ. Online* 14 815–824. 10.17051/io.2015.03160

[B2] Aluja-Banet T. Nafria E. (2003). Stability and scalability in decision trees. *Comput. Stat.* 18 505–520. 10.1007/BF03354613

[B3] Asensio I. Carpintero E. Expósito E. López E. (2018). How much gold is in the sand? Data mining with spain’s PISA 2015 results. *Revista Española de Pedagogía* 76 225–245.

[B4] Asif R. Merceron A. Ali S. A. Haider N. G. (2017). Analyzing undergraduate students’ performance using educational data mining. *Comput. Educ.* 113 177–194. 10.1016/j.compedu.2017.05.007

[B5] Bronfenbrenner U. (1979). *The Ecology of Human Development: Experiments by Nature and Design.* Cambridge: Harvard University Press.

[B6] Bryk A. S. Raudenbush S. W. (1992). *Hierarchical Linear Models: Applications and Data Analysis Methods.* Newbury Park: Sage Publications.

[B7] Casey K. Azcona D. (2017). Utilizing student activity patterns to predict performance. *Int. J. Educ. Technol. High. Educ.* 14:4 10.1186/s41239-017-0044-3

[B8] Chapman C. Muijs D. Raynolds D. Sammons P. Teddlie C. (eds). (2016). *The Routledge International Handbook of Educational Effectiveness and Improvement Research, Policy, and Practice.* New York, NY: Routledge.

[B9] Coleman J. S. Campbell E. Q. Hobson C. J. McPartland J. Mood A. M. Weinfeld F. D. (1966). *Equality of Educational Opportunity (N.o OE-38001).* Washington, DC: National Center for Educational Statistics.

[B10] Cordero J. M. Pedraja F. Simancas R. (2015). Success factors for educational attainment in unfavorable socioeconomic conditions. *Revista de Educacion* 2015 163–187. 10.4438/1988-592X-RE-2015-370-302 6239832

[B11] Costa E. B. Fonseca B. Almeida M. Ferreira F. Rego J. (2017). Evaluating the effectiveness of educational data mining techniques for early prediction of students’ academic failure in introductory programming courses. *Comput. Hum. Behav.* 73 247–256. 10.1016/j.chb.2017.01.047

[B12] Costa P. Araújo L. (2017). Skilled students and effective schools: reading achievement in denmark, sweden, and france. *Scand. J. Educ. Res.* 62 850–864. 10.1080/00313831.2017.1307274

[B13] Creemers B. Kyriakides L. (2015). Developing, testing, and using theoretical models for promoting quality in education. *Sch. Eff. Sch. Improv.* 26 102–119. 10.1080/09243453.2013.869233 10151842

[B14] Creemers B. Scheerens J. (1994). Developments in the educational effectiveness research programme. *Int. J. Educ. Res.* 21 125–140. 10.1016/0883-0355(94)90028-0

[B15] Fernandez-Cano A. (2016). A methodological critique of the PISA evaluations. *RELIEVE* 22:Art M15 10.7203/relieve.22.1.8806

[B16] Gamazo A. Martínez-Abad F. Olmos-Migueláñez S. Rodríguez-Conde M. J. (2018). Assessment of factors related to school effectiveness in PISA 2015. a multilevel analysis. *Revista de Educacion* 2017 56–84. 10.4438/1988-592X-RE-2017-379-369 31379678

[B17] Goldstein H. (1995). *Multilevel Statistical Models.* Oxford: Oxford University Press.

[B18] González C. Caso J. Díaz K. López M. (2012). Rendimiento académico y factores asociados: aportaciones de algunas evaluaciones a gran escala. Bordón. *Revista de pedagogía* 64 51–68.

[B19] González-Such J. Sancho-Álvarez C. Sánchez-Delgado P. (2016). Background questionnaires of PISA: a study of the assessment indicators. *RELIEVE* 22:M7 10.7203/relieve.22.1.8274

[B20] Guruler H. Istanbullu A. Karahasan M. (2010). A new student performance analysing system using knowledge discovery in higher educational databases. *Comput. Educ.* 55 247–254. 10.1016/j.compedu.2010.01.010

[B21] Hopfenbeck T. N. Lenkeit J. El M. Cantrell K. Ryan J. Baird J.-A. (2018). Lessons learned from PISA: a systematic review of peer-reviewed articles on the programme for international student assessment. *Scand. J. Educ. Res.* 62 333–353. 10.1080/00313831.2016.1258726

[B22] Hox J. (2010). *Multilevel Analysis: Techniques and Applications*, 2nd Edn New York, NY: Routledge.

[B23] Huang X. Wilson M. Wang L. (2016). Exploring plausible causes of differential item functioning in the PISA science assessment: language, curriculum or culture. *Educ. Psychol.* 36 378–390. 10.1080/01443410.2014.946890

[B24] Hung J. L. Hsu Y. C. Rice K. (2012). Integrating data mining in program evaluation of K-12 online education. *Educ. Technol. Soc.* 15 27–41.

[B25] Jacobucci R. (2018). Decision tree stability and its effect on interpretation. *PsyArXiv* [Preprint].

[B26] Jamain A. Hand D. J. (2008). Mining supervised classification performance studies: a meta-analytic investigation. *J. Classif.* 25 87–112. 10.1007/s00357-008-9003-y

[B27] Joaristi L. Lizasoain L. Azpillaga V. (2014). Detection and characterization of highly effective schools in the autonomous community of the basque country using contextualized cross-sectional attainment models and hierarchical linear models. *Estudios Sobre Educacion* 27 37–61. 10.15581/004.27.37-61

[B28] Jornet M. (2016). Methodological analysis of the PISA project as international assessment. *RELIEVE* 22:Art.M1 10.7203/relieve22.1.8293

[B29] Kasih J. Ayub M. Susanto S. (2013). Predicting students’ final passing results using the classification and regression trees (cart) algorithm. *World Trans. Eng. Technol. Educ.* 11 46–49.

[B30] Kieskamp M. H. (2015). Please Fasten Your Seat Belts. Data Analysis Towards the Origin of Departure Delay of KLM Cityhopper at Schiphol Airport. Available at: https://essay.utwente.nl/68918/1/Kieskamp_MA_BMS.pdf (accessed August 19, 2019).

[B31] Kılıç D. As̨kın ÖE. Öz E. (2017). Identifying the classification performances of educational data mining methods: a case study for TIMSS. *Kuram ve Uygulamada Egitim Bilimleri* 17 1605–1623. 10.12738/estp.2017.5.0634

[B32] Kirby N. F. Dempster E. R. (2014). Using decision tree analysis to understand foundation science student performance. Insight gained at one south african university. *Int. J. Sci. Edu.* 36 2825–2847. 10.1080/09500693.2014.936921

[B33] Landwehr N. Hall M. Frank E. (2005). Logistic model trees. *Mach. Learn.* 59 161–205. 10.1007/s10994-005-0466-3

[B34] Lavy V. (2015). Do differences in schools’ instruction time explain international achievement gaps? Evidence from developed and developing countries. *Econ. J.* 125 F397–F424. 10.1111/ecoj.12233 14986321

[B35] Lenkeit J. Caro D. H. (2014). Performance status and change—measuring education system effectiveness with data from PISA 2000-2009. *Educ. Res. Eval.* 20 146–174. 10.1080/13803611.2014.891462

[B36] Lezotte L. W. (1989). School improvement based on the effective schools research. *Int. J. Educ. Res.* 13 815–825. 10.1016/0883-0355(89)90031-1

[B37] Liu H. Motoda H. (1998). *Feature Selection for Knowledge Discovery and Data Mining.* Norwell, MA: Kluwer Academic Publishers.

[B38] Liu X. Ruiz M. E. (2008). Using data mining to predict K-12 students’ performance on large-scale assessment items related to energy. *J. Res. Sci. Teach.* 45 554–573. 10.1002/tea.20232

[B39] Liu X. Whitford M. (2011). Opportunities-to-learn at home: profiles of students with and without reaching science proficiency. *J. Sci. Educ. Technol.* 20 375–387. 10.1007/s10956-010-9259-y

[B40] Lopez-Agudo L. A. Jerrim J. Marcenaro-Gutierrez O. D. Shure N. (2017). “To weight or not to weight? The case of PISA data,” in *Investigaciones de Economía de la Educación*, eds ómez Gallego J. C. G. érez Cárceles M. C. P. Nieto Torrejón L. (Madrid: Asociación de Economía de la Educación).

[B41] Martínez R. Gaviria J. L. Castro M. (2009). Concept and evolution of educational value-added models. *Revista de Educacion* 348 15–45.

[B42] Martínez-Abad F. Chaparro-Caso-López A. A. (2017). Data-mining techniques in detecting factors linked to academic achievement. *Sch. Eff. Sch. Improv.* 28 39–55. 10.1080/09243453.2016.1235591

[B43] Martínez-Abad F. Gamazo A. Rodríguez-Conde M. J. (2018). Big data in education: detection of ICT factors associated with school effectiveness with data mining techniques. *Proc. TEEM* 18 145–150. 10.1145/3284179.3284206

[B44] Martínez-Garrido C. Murillo F. J. (2016). Investigación iberoamericana sobre enseñanza eficaz. *Revista mexicana de investigación educativa* 21 471–499.

[B45] McNeish D. M. Stapleton L. M. (2016). The effect of small sample size on two-level model estimates: a review and illustration. *Educ. Psychol. Rev.* 28 295–314. 10.1007/s10648-014-9287-x

[B46] Mitchell A. J. (2009). A meta-analysis of the accuracy of the mini-mental state examination in the detection of dementia and mild cognitive impairment. *J. Psychiatr. Res.* 43 411–431. 10.1016/j.jpsychires.2008.04.014 18579155

[B47] Muijs D. Kyriakides L. Werf G. van der Creemers B. Timperley H. Earl L. (2014). State of the art – teacher effectiveness and professional learning. *Sch. Eff. Sch. Imp.* 25 231–256. 10.1080/09243453.2014.885451

[B48] Murillo J. (2007). “School effectiveness research in latin america,” in *International Handbook of School Effectiveness and Improvement*, ed. Townsend T. (Netherlands: Springer), 75–92. 10.1007/978-1-4020-5747-2_5

[B49] OECD, (2017). *PISA 2015. Technical Report.* París: OECD Publishing.

[B50] OECD, (2019). *“PISA: Programme for International Student Assessment”, OECD Education Statistics (database).* París: OECD Publishing.,

[B51] Oskouei R. J. Askari M. (2014). Predicting academic performance with applying data mining techniques (generalizing the results of two different case studies). *Comput. Eng. Appl. J.* 3 79–88. 10.18495/comengapp.v3i2.81

[B52] Pitsia V. Biggart A. Karakolidis A. (2017). The role of students’ self-beliefs, motivation and attitudes in predicting mathematics achievement a multilevel analysis of the programme for international student assessment data. *Learn. Individ. Differ.* 55 163–173. 10.1016/j.lindif.2017.03.014

[B53] Quinlan J. R. (1986). Induction of decision trees. *Mach. Learn.* 1 81–106. 10.1023/A:1022643204877

[B54] Quinlan R. (1992). *C4.5: Programs for Machine Learning. San Mateo.* California: Morgan Kaufmann Publishers Inc.

[B55] Raudenbush S. W. Bryk A. S. Cheong Y. F. Congdon R. T. Du Toit M. (2016). *HLM7 Hierarchical Linear and Nonlinear Modeling User Manual: User Guide for Scientific Software International’s (S.S.I.) Program*, Edn 1 Skokie, IL: Scientific Software International, Incorporated.

[B56] Raudenbush S. W. Willms J. D. (1995). The estimation of school effects. *J. Educ. Behav. Stat.* 20 307–335. 10.3102/10769986020004307

[B57] Roberts J. K. Monaco J. P. Stovall H. Foster V. (2011). “Explained variance in multilevel models,” in, *Handbook of Advanced Multilevel Analysis* eds Hox J. Roberts J. K. (New York, NY: Routledge), 219–230.

[B58] Rodrigues M. W. Isotani S. Zárate L. E. (2018). Educational data mining: a review of evaluation process in the e-learning. *Telem. Inform.* 35 1701–1717. 10.1016/j.tele.2018.04.015

[B59] Romero C. López M. I. Luna J.-M. Ventura S. (2013). Predicting students’ final performance from participation in on-line discussion forums. *Comput. Educ.* 68 458–472. 10.1016/j.compedu.2013.06.009

[B60] Rutkowski L. Rutkowski D. (2010). Getting it ‘better’: the importance of improving background questionnaires in international large-scale assessment. *J. Curriculum Stud.* 42 411–430. 10.1080/00220272.2010.487546

[B61] Rutter M. Maughan B. (2002). School effectiveness findings 1979–2002. *J. Sch. Psychol. J. Sch. Psychol.* 40 451–475. 10.1016/S0022-4405(02)00124-3

[B62] S̨ara N.-B. Halland R. Igel C. Alstrup S. (2015). “High-school dropout prediction using machine learning: a danish large-scale study,” in *Proceedings of the European Symposium on Artificial Neural Networks, (ESANN 2015)*, (Bruges: Computational Intelligence and Machine Learning), 319–324.

[B63] Tan C. Y. Hew K. F. (2017). Information technology, mathematics achievement and educational equity in developed economies. *Educ. Stud.* 43 371–390. 10.1080/03055698.2016.1277137

[B64] Tan M. Shao P. (2015). Prediction of student dropout in E-learning program through the use of machine learning method. *Int. J. Emerg. Technol. Learn.* 10 11–17. 10.3991/ijet.v10i1.4189

[B65] Turney P. D. (1995). Technical note: bias and the quantification of stability. *Mach. Learn.* 20 23–33. 10.1007/BF00993473

[B66] Wrigley T. (2013). Rethinking school effectiveness and improvement: a question of paradigms. *Discourse* 34 31–47. 10.1080/01596306.2012.698862

[B67] Xu Y. J. (2005). An exploration of using data mining in educational research. *J. Mod. Appl. Stat. Methods* 4 251–274. 10.22237/jmasm/1114906980

[B68] Yu X. Shi Y. Zhang L. Nie G. Huang A. (2014). Intelligent knowledge beyond data mining: influences of habitual domains. *Commun.Assoc. Inform. Sys.* 34 985–1000. 10.17705/1CAIS.03453

